# Income or Job Loss and Psychological Distress During the COVID-19 Pandemic

**DOI:** 10.1001/jamanetworkopen.2024.24601

**Published:** 2024-07-30

**Authors:** Grace V. Ringlein, Catherine K. Ettman, Elizabeth A. Stuart

**Affiliations:** 1 Department of Biostatistics, Johns Hopkins Bloomberg School of Public Health, Baltimore, Maryland; 2 Department of Health Policy and Management, Johns Hopkins Bloomberg School of Public Health, Baltimore, Maryland

## Abstract

**Importance:**

Given the expiration of expanded unemployment and other benefits during the COVID-19 pandemic, it is important to understand the association between pandemic income or job loss and long-term implications on mental health.

**Objective:**

To evaluate the association between income or job loss due to the COVID-19 pandemic and later psychological distress.

**Design, Setting, and Participants:**

This cohort study used 5 waves of nationally representative, longitudinal survey data (September 16, 2019, through September 18, 2022) from the Pew Research Center’s American Trends Panel. Doubly robust propensity score–weighted quasi-Poisson models were used to estimate the association of self or household income or job loss during the early phase of the COVID-19 pandemic with later psychological distress, while controlling for pre–income loss characteristics (demographics, finances, and psychological distress). The study sample comprised US working-age adults (aged 18-64 years) who had not experienced income or job loss due to the COVID-19 pandemic by March 24, 2020.

**Exposure:**

Participant-reported self or household income or job loss (ie, reduced hours or demand for work) due to the COVID19 pandemic between March 24, 2020, and August 16, 2020.

**Main Outcome and Measures:**

Psychological distress was measured using a composite scale of 0 to 15 based on participants’ reported frequency of feeling depressed, on edge, sleepless, lonely, and hopeless in the past week in March 2020, February 2021, and September 2022.

**Results:**

Of 1392 working-age adults (survey weighted 52.7% male and 47.7% aged 30-49 years) who had not reported income or job loss before March 24, 2020, a survey weighted 35.7% reported job or income loss between March 24 and August 16, 2020. Early-phase pandemic income or job loss was associated with higher distress in February 2021 (estimated ratio, 1.09; 95% CI, 1.01-1.18; *P* = .03) and September 2022 (estimated ratio, 1.11; 95% CI, 1.02-1.22; *P* = .02) among participants who experienced job or income loss between March 24 and August 16, 2020, compared with the propensity score–weighted mean in the group who did not experience income loss.

**Conclusions and Relevance:**

These small but significant within-person associations between early-phase pandemic household income or job loss and psychological distress up to 29 months later suggest that policies are needed to support people with income or job loss to help mitigate the long-term adverse mental health outcomes of economic disruption.

## Key Points

**Question:**

What is the association between income or job loss in the early phase of the COVID-19 pandemic and later psychological distress?

**Findings:**

In this cohort study of 1392 US working-age adults, income or job loss between March 24 and August 16, 2020, was associated with psychological distress scores that were 1.09 and 1.11 times higher in February 2021 and September 2022, respectively, compared with individuals with no income or job loss.

**Meaning:**

These findings suggest the need for policies to support people with income or job loss to help mitigate long-term adverse mental health outcomes of economic disruption.

## Teaser

This cohort study evaluates the association between income or job loss due to the COVID-19 pandemic and later psychological distress in US working-age adults.

## Introduction

The start of the COVID-19 pandemic brought record levels of unemployment, which were above 14% by April 2020,^1,2^ and a worsening of mental health^3,4,5,6,7^ across the US compared with before the pandemic. The burden of pandemic job loss was felt unequally, with younger; less-educated; Asian, Black, and Hispanic; female; and low-wage workers more likely to lose their jobs.^1,8^ Although employment returned to prepandemic rates by June 2020 for higher-wage workers, employment remained down by 13% for lower-wage workers in December 2021.^2^ Additionally, adults with lower income and less savings were at higher risk of early pandemic depression.^9^

Associations between unemployment or income loss and poor mental health in the US have been documented during prior global economic crises, such as the Great Recession,^10,11,12^ nonrecessionary periods,^13,14,15^ and the early COVID-19 pandemic, in both cross-sectional studies^16,17,18,19,20,21,22,23^ and longitudinal cohort studies.^24,25^ However, use of standard regression adjustment methods to estimate associations may not fully account for differences between exposure groups that relate to both their probability of experiencing job loss and having worse mental health. Well-established statistical methods could be used to account for these factors and generate causal estimates in observational studies, contingent on the validity of the assumptions, such as no unobserved confounders.^26,27,28^ Prior to the pandemic, studies that aimed to estimate causal effects have supported a significant association between job loss and worse mental health both in other countries^29,30,31,32^ and the US,^14,33,34^ although 1 study did not show significant differences.^35^

Among studies comparing job loss with mental health outcomes during the pandemic that used methods aimed at estimating causal effects, we identified 3 in US working-age adult populations.^21,25,36^ Using targeted maximum likelihood estimation, Lee et al^25^ estimated a significant association between job insecurity in April 2020 and mental health in May 2020 in a population-based longitudinal sample. Wu et al^21^ reported a significant association between job loss and poor mental health, using propensity score weighting in a cross-sectional survey of custodial grandparents conducted in June 2020. Using an instrumental variable and bounding approach, Baird et al^36^ did not find evidence of a significant causal effect of unemployment on psychological distress in a sample of African American adults living in Pittsburgh, Pennsylvania, in 2020. Given the limited number of studies that used methods to account for differences in the likelihood of job loss between exposure groups, with only 1 focused on the general US working-age population,^25^ and the inconsistency in the results, more research is needed. Additionally, the expiration of enhanced unemployment and other financial benefits by September 2021^37,38,39^ has called for evidence on the long-term influence of pandemic-era stressors on mental health. Thus, our work investigates the association between income loss early in the COVID-19 pandemic and mental health more than 2 years later between US working-age adults (aged 18-64 years) who did and did not report income or job loss.

## Methods

### Data and Sample

In this cohort study, we used the Pew Research Center’s American Trends Panel (ATP), a nationally representative, longitudinal survey from which subsamples of participants are invited to complete repeated waves of data collection.^40^ Because the ATP consists of publicly available, nonidentifiable data, this study was classified by the Johns Hopkins Bloomberg School of Public Health institutional review board as non–human participant research. This study followed the Strengthening the Reporting of Observational Studies in Epidemiology (STROBE) reporting guideline.

We used data from wave 54 (September 16-29, 2019), wave 64 (March 19-24, 2020), wave 72 (August 3-16, 2020), wave 83 (February 16-21, 2021), and wave 114 (September 13-18, 2022) of the ATP. Participants reported on psychological distress in waves 64, 83, and 114; pandemic income or job in waves 64 and 72; lifetime diagnosis of a mental illness in wave 64; and prepandemic finances in wave 54. Demographic information came from waves 54 and 64.

Our main analysis included adults aged 18 to 64 years with data from all 5 waves who were eligible for job loss analysis between March 24 and August 16, 2020. This group (1473 participants) included adults who did not report household income or job loss prior to March 24 and did not report that job loss in wave 72 (August 3-16, 2020) was not applicable. For this cohort, the measurement of baseline psychological distress (in March 2020) was reported prior to income or job loss.

Where possible, missing demographic data and income tiers were imputed using data from the closest nonmissing time point. Following that imputation, we conducted a complete case analysis, excluding those missing data on any of the other variables of interest (81 participants [5.5%]). The eMethods in Supplement 1 provide additional details on survey methods, sample inclusion, and treatment of missing data. Characteristics of the participants excluded for missing data are provided in eTable 1 in Supplement 1, and sample construction is illustrated in eFigure 1 in Supplement 1. Our analytic sample consisted of US working-age adults who did not experience household income or job loss due to the pandemic by March 24, 2020. The Pew Research Center created customized survey weights (additional details provided in the eMethods in Supplement 1), which accounted for nonresponse and aligned the sample with the US population of adults aged 18 to 64 years who did not experience income or job loss prior to March 24, 2020.

### Measures

#### Income or Job Loss Due to the Pandemic

Our exposure of interest was income or job loss early in the pandemic. We defined income or job loss by whether the participant reported that they or someone in their household was laid off or lost a job or had to take a cut in pay due to reduced hours or demand for work due to the COVID-19 pandemic. We defined early-phase pandemic income or job loss as having occurred between March 24, 2020, and August 16, 2020, but not before.

#### Pandemic Psychological Distress

We use a composite measure of psychological distress, based on the Center for Epidemiological Studies Depression Scale and Generalized Anxiety Disorder 7-item,^41,42^ as previously published.^43,44^ In March 2020, February 2021, and September 2022, participants were asked to rate the frequency with which they experienced the following 5 conditions in the past 7 days: felt nervous, anxious, or on edge; felt depressed; felt hopeful about the future; felt lonely; and had trouble sleeping. Each domain was rated on a 4-value scale ranging from rarely or none of the time (<1 day) to most or all of the time (5-7 days), which we coded as 0 to 3 (with hopefulness reverse-coded to represent lack of hope). Summing across the 5 domains, we computed the psychological distress score (possible range, 0-15).

#### Preexposure Covariates

We use the following covariates in the propensity score model and quasi-Poisson models: demographics (age, sex, race and ethnicity, marital status, parental status, US citizenship, census region, metropolitan area indicator, education level), prepandemic financial status (income tier, financial strain, savings account, secured debt, unsecured debt, employment status), psychological distress before income or job loss, and lifetime diagnosis of a mental health condition. We included race and ethnicity as a covariate in our analyses, as previous work has shown race and ethnicity to be associated both with the probability of pandemic job loss^1,8^ and mental health.^25^ Participants self-reported race and ethnicity by selecting 1 of the following 4 categories presented on the ATP questionnaire in March 2020: Hispanic, non-Hispanic Black (hereafter Black), non-Hispanic White (hereafter White), and other. While the other category did not explicitly state the groups included, future iterations of the ATP broke this group into 3 categories: Asian or Asian American, mixed race, or some other race. Additional details about the measures are provided in the eMethods in Supplement 1.

### Statistical Analysis

First, we presented survey-weighted descriptive statistics of our analytic sample using the gtsummary package in R.^45^ We quantified the observed differences between participants who reported income or job loss and those who did not using standardized mean differences (SMDs) and survey-weighted χ^2^ tests with Rao-Scott second-order correction^46^ for categorical covariates and Wilcoxon rank sum tests for complex survey samples^47^ for pre–income loss psychological distress in March 2020.

Second, to understand and account for differences between the exposure groups, we computed propensity scores, an estimate of the probability of income or job loss, using survey-weighted logistic regression with the WeightIt package in R.^48^ We included all preexposure covariates discussed above in the propensity score model. Given our interest in understanding the mental health of participants who experienced early-phase pandemic income or job loss, these individuals were assigned propensity score weights of 1, and those who did not experience income or job loss were weighted by their estimated odds of exposure.^28^ We assessed whether the balance after weighting was acceptable by looking at SMDs. We graphically examined the distribution of propensity scores and weights to check for common support (sufficient overlap over the range of propensity scores in the 2 groups) and to identify any extreme weights.^28,49^

Third, for our main analysis, we used propensity score– and survey-weighted quasi-Poisson models (log-link), implemented with the survey package in R,^50^ to investigate how early-phase pandemic income or job loss compared with later pandemic mental health. The eMethods in Supplement 1 include details on model choice, and the eResults in Supplement 1 contain a supplemental analysis using an ordinary least squares model instead of a quasi-Poisson model.

We controlled for preexposure psychological distress (March 2020) and demographic and financial covariates through the regression model and through propensity score weighting, providing a doubly robust estimate.^51,52^ As our primary finding, we report the ratio of the expected psychological distress score in the income or job loss group relative to the expected score in the group that did not report income or job loss, weighted by their estimated odds of exposure. The estimates were obtained by exponentiating the income or job loss coefficient from the models and are presented with 95% CIs. To aid interpretation, we also computed the estimates as differences in means (using the marginaleffects package in R).^53^ Separate models were fit for psychological distress in February 2021 and September 2022.

Fourth, to assess how sensitive our results could be to an unobserved confounder, we estimated the E-value (using the EValue package in R^54^), or the minimum association required between both the confounder and income or job loss and between the confounder and psychological distress, to explain any observed associations.^54^

The statistical analyses were performed using R, version 4.3.1 software (R Foundation for Statistical Computing). All statistical tests were 2-sided, with an α = .05 significance level.

## Results

### Descriptive Statistics

The Table shows the demographic characteristics of the sample. A total of 1392 participants comprised the full analytic sample, of whom a weighted 35.7% reported early-phase pandemic household income or job loss. The unadjusted sample was 47.3% female and 52.7% male; 47.7% were aged 30-49 years; 11.8% were Black, 15.6% Hispanic, 63.0% White, and 9.6% other race and ethnicity; 55.9% were married; and 50.9% were in the middle-income tier. Individuals who did and did not report income loss were generally similar with respect to the observed covariates and pre–income loss psychological distress, with significant differences observed, respectively, in age (18-29 years, 29.8% vs 21.7%; 30-49 years, 46.1% vs 48.5%; 50-64 years, 24.1% vs 29.8%; *P* = .04), race and ethnicity (Black, 10.2% vs 12.7%; Hispanic, 20.4% vs 13.0%; White, 57.7% vs 65.9%; other, 11.6% vs 8.4%; *P* = .04), census region (South, 38.6% vs 40.5%; Northeast, 12.2% vs 18.7%; Midwest, 20.2% vs 20.8%; West, 29.0% vs 20.1%; *P* = .02), and prepandemic income tier (upper, 18.4% vs 26.1%; lower, 31.5% vs 22.5%, middle, 50.1% vs 51.4%; *P* = .01). Propensity score weighting adjusts for such differences in our analyses.

**Table.** Analytic Sample Characteristics^a^

Characteristic	No. of participants (weighted %)^b^			*P* value^c^
	Full analytic sample	Early-phase pandemic income or job loss	No pandemic income or job loss	
Full analytic sample	1392 (100)	437 (35.7)	955 (64.2)	NA
Sex				
Female	698 (47.3)	220 (44.5)	478 (48.9)	.25
Male	694 (52.7)	217 (55.5)	477 (51.1)	
Age category, y				
18-29	258 (24.6)	95 (29.8)	163 (21.7)	.04
30-49	638 (47.7)	197 (46.1)	441 (48.5)	
50-64	496 (27.8)	145 (24.1)	351 (29.8)	
Race and ethnicity				
Black, non-Hispanic	130 (11.8)	40 (10.2)	90 (12.7)	.04
Hispanic	188 (15.6)	68 (20.4)	120 (13.0)	
White, non-Hispanic	971 (63.0)	289 (57.7)	682 (65.9)	
Other^d^	103 (9.6)	40 (11.6)	63 (8.4)	
Marital status				
Married	807 (55.9)	256 (54.6)	551 (56.6)	.77
Divorced or separated	144 (9.6)	49 (9.4)	95 (9.6)	
Living with a partner	125 (10.1)	41 (12.0)	84 (9.1)	
Widowed	28 (1.2)	9 (1.3)	19 (1.2)	
Never been married	288 (23.2)	82 (22.7)	206 (23.5)	
Education category				
Less than high school	31 (5.5)	10 (6.7)	21 (4.8)	.38
High school graduate	201 (21.9)	59 (24.1)	142 (20.7)	
Associate’s degree	138 (9.3)	39 (6.6)	99 (10.7)	
Some college	295 (24.2)	99 (25.1)	196 (23.7)	
College graduate or higher	421 (22.6)	142 (22.5)	279 (22.7)	
Postgraduate	306 (16.5)	88 (15.0)	218 (17.4)	
Parent of a child aged <18 y	489 (35.8)	164 (36.8)	325 (35.3)	.69
US citizenship	1350 (94.2)	420 (93.4)	930 (94.7)	.49
Census region				
South	524 (39.8)	164 (38.6)	360 (40.5)	.02
Northeast	221 (16.4)	58 (12.2)	163 (18.7)	
Midwest	317 (20.6)	95 (20.2)	222 (20.8)	
West	330 (23.3)	120 (29.0)	210 (20.1)	
Metropolitan area	1234 (87.3)	393 (88.4)	841 (86.7)	.54
Income tier (prepandemic)				
Upper	415 (23.3)	108 (18.4)	307 (26.1)	.01
Lower	251 (25.7)	99 (31.5)	152 (22.5)	
Middle	726 (50.9)	230 (50.1)	496 (51.4)	
Financial strain (prepandemic)	450 (37.7)	158 (41.7)	292 (35.5)	.11
Employment status (prepandemic)				
Full time	976 (64.9)	301 (63.7)	675 (65.5)	.10
Part time	163 (12.8)	60 (16.3)	103 (10.8)	
Not employed	253 (22.4)	76 (20.0)	177 (23.7)	
Had a savings account (prepandemic)	1130 (74.7)	349 (71.9)	781 (76.3)	.25
Had secured debt (prepandemic)	989 (65.4)	306 (62.8)	683 (66.8)	.31
Had unsecured debt (prepandemic)	941 (68.1)	303 (69.9)	638 (67.1)	.46
Lifetime diagnosis of a mental health condition	222 (16.0)	72 (15.4)	150 (16.4)	.72
Psychological distress in March 2020, mean (SE)^e^^,^^f^	5.1 (0.1)	5.1 (0.2)	5.1 (0.2)	.69^g^

Abbreviation: NA, not applicable.

^a^ The analytic sample consisted of US adults aged 18 to 64 years who did not experience self or household income or job loss before March 24, 2020, based on the Pew Research Center’s American Trends Panel.

^b^ Using longitudinal survey weights.

^c^ *P* values are reported for χ^2^ tests with Rao-Scott second-order correction, comparing the sample proportions of covariates between participants who experienced early-phase pandemic income or job loss and those who did not experience income or job loss. One value is reported per covariate.

^d^ The other category did not explicitly state the groups included. Future iterations of the American Trends Panel broke this group into 3 categories: Asian or Asian American, mixed race, or some other race.

^e^ Mean and standard error are weighted using longitudinal survey weights.

^f^ Psychological distress measured on a composite scale of 0 to 15 based on participants’ reported frequency of feeling depressed, on edge, sleepless, lonely, and hopeless in the past week. Psychological distress in March 2020 is reported prior to any of the sample experiencing household income or job loss.

^g^ *P* value is reported for a Wilcoxon rank sum test for complex survey samples, comparing mean psychological distress scores between income or job loss groups.

### Propensity Score Weighting

**Figure 1.**
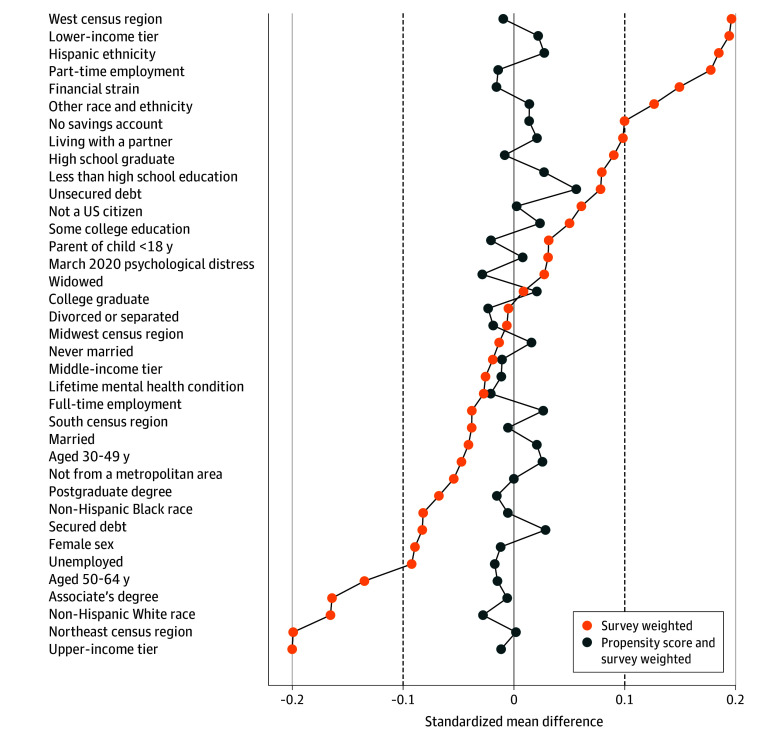
Survey-Weighted Covariate Balance Before and After Propensity Score Weighting Data are from the Pew Research Center’s American Trends Panel, and the sample included 1392 US adults aged 18 to 64 years who had not experienced income or job loss by March 24, 2020. Standardized mean differences were computed for each covariate group, before propensity score adjustment (using only survey weights) and after propensity score weighting (using both survey weights and propensity score weights), comparing the covariate balance between the group that experienced early-phase pandemic income loss and the group that did not experience pandemic income loss. The dashed lines at −0.1 and 0.1 indicate a cutoff for acceptable balance defined in Austin.^49^

Figure 1 shows SMDs in the covariates between groups. After propensity score weighting, the SMD for all covariates was below a cutoff of 0.1, as used by Austin^49^ to determine acceptable balance, and the balance on preexposure psychological distress was good, indicating no substantial differences in psychological distress before the job or income loss. eTable 2 in Supplement 1 presents the details of the multivariable logistic regression model used to estimate propensity scores and the SMD for each covariate. The distributions of propensity scores (eFigure 2 in Supplement 1) and weights (eFigure 3 in Supplement 1) show sufficient overlap across groups and no extreme weights (discussed further in the eMethods in Supplement 1).

### Associations Between Early-Phase Pandemic Income Loss and Later Psychological Distress

**Figure 2.**
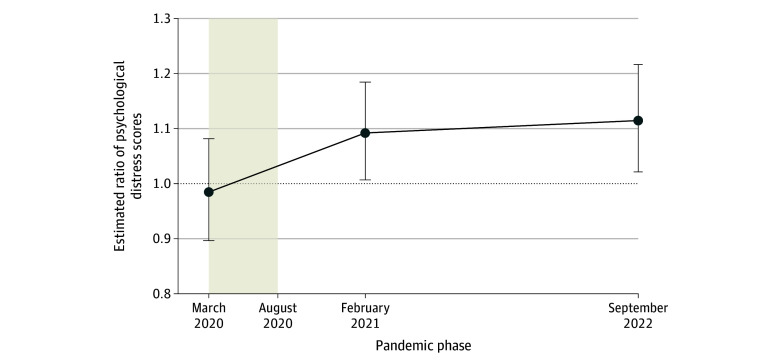
Adjusted Ratio of Psychological Distress Scores for Early-Phase Pandemic Income or Job Loss Data are from the Pew Research Center’s American Trends Panel, and the sample included 1392 US adults aged 18 to 64 years who had not experienced income or job loss by March 24, 2020. Points indicate the adjusted ratio of psychological distress scores and whiskers the 95% CI for individuals who experienced early-phase pandemic income loss compared with the propensity score–weighted mean in the group that did not experience income loss. This ratio was estimated using covariate-adjusted, survey-weighted, and propensity score–weighted quasi-Poisson models. Psychological distress was measured on a composite scale of 0 to 15 based on participants’ reported frequency of feeling depressed, on edge, sleepless, lonely, and hopeless in the past week reported in February 2021 and September 2022. The shaded bar represents the time (March 24 to August 16, 2020) during which the early-phase pandemic income or job loss occurred, and the dashed line indicates the null hypothesis that the ratio equals 1.

We estimated that early-phase pandemic income or job loss was associated with higher psychological distress in February 2021 (estimated ratio, 1.09; 95% CI, 1.01-1.18; *P* = .03) and September 2022 (estimated ratio, 1.11; 95% CI, 1.02-1.22; *P* = .02) compared with the propensity score–weighted mean in the group that did not experience income loss (Figure 2). On a difference-in-means scale, these estimates translate to an increase of 0.42 points (95% CI, 0.03-0.80 points; *P* = .03) and 0.52 points (95% CI, 0.10-0.95 points; *P* = .02), respectively (eTable 3 in Supplement 1). Analogous results where an ordinary least squares model was used instead of the quasi-Poisson model are reported in the eResults in Supplement 1, with similar substantive results.

### Sensitivity to an Unobserved Confounder

We estimated that an unobserved confounder would need to be associated with both income or job loss and psychological distress by 1.41-fold in February 2021 and by 1.47-fold September 2022 to explain away our estimated ratios of 1.09 and 1.11. Although it is possible that such a confounder exists, we feel confident that through controlling for demographics, prepandemic finances, and lifetime diagnosis of a mental health condition in our model, we account for most conceivable potential confounders.

## Discussion

In this nationally representative, longitudinal cohort study of working-age adults using doubly robust regression^51,52^ analysis with propensity score weighting, we found that self or household income or job loss between March 24 and August 16, 2020, was associated with 9% and 11% higher psychological distress 6 to 10 and 25 to 29 months later, respectively. These findings highlight the necessity of studying the long-term mental health of individuals who experience income or job loss.

We conservatively refer to the parameter of interest as an association. Contingent on the validity of assumptions, our estimates could be interpreted as the mean effect of early-phase pandemic income loss or job loss on later psychological distress for those who experienced it.^28,51^ These assumptions include common support and that there are no confounders associated with both early-phase pandemic income or job loss and mental health beyond those we adjusted for in our models.

Our results are generally consistent with the few studies that have used causal methods to study the association between pandemic household income or job loss and mental health. Using propensity score weighting with a sample of custodial grandparents, Wu et al^21^ reported those who lost jobs had a mean score on the Mental Health Inventory-5 (1-6 scale) in June 2020 that was 0.26 points worse than those who did not experience job loss. Lee et al^25^ reported an adjusted odds ratio of 1.66 for depression and 1.50 for anxiety for individuals who experienced unemployment or underemployment compared with those working full time, using a targeted maximum likelihood estimation with longitudinal data collected between April and May 2020.

To situate our findings in the broader literature on job loss and mental health, it is necessary to consider particularities about income or job loss that occurred due to the COVID-19 pandemic that may have informed the mental health outcomes associated with income or job loss. First, a larger proportion of workers who experienced job loss expected to be rehired, and fewer were looking for new jobs, compared with previous economic recessions.^1^ Second, the passage of federal and state policies resulting in the most generous financial support to date^37^ may have weakened the association of income loss on psychological distress. Economic impact payments were distributed in March 2020, December 2020, and March 2021,^39^ and the Coronavirus Aid, Relief, and Economic Security Act, signed on March 27, 2020, funded programs that provided extended unemployment benefits via supplemental payments, extended the number of weeks of benefits, and provided additional eligibility for benefits.^37,38^ Evidence that receipt of unemployment benefits may have weakened the association between unemployment and worse mental health due to the pandemic^14,16^ may explain the slightly smaller association in our sample than in other studies of adults that used different time frames. Third, the psychological effects of mass recession may differ from more isolated job loss. Given previous evidence on potential social comparison,^55^ it is possible that large-scale job loss may have different associations than off-cycle job loss, which can be felt more personally. Fourth, while we estimated within-person changes in mental health, we compared individuals who did and did not experience job loss. Given the high rates of psychological distress among the whole population, including employed people, it is possible that the association between job loss and mental health during the pandemic was smaller than may be expected in other periods.

### Limitations

Our findings should be considered in the context of 4 limitations. First, our analysis does not include adults reporting income or job loss before March 24, 2020; thus, our results do not reflect the subset of the population who experienced income loss in the earliest portion of the pandemic, which may consist of individuals with greater economic precarity.^1,2^ Second, we do not know whether any of the individuals who did not experience income loss by August 2020 experienced income loss due to the pandemic after that point. Third, although we adjusted for a broad set of pre–income or job loss characteristics, there may be an unobserved confounder that explains the observed associations. For example, due to data limitations, we were unable to control for occupation type or receipt of unemployment benefits. It is possible that more generous economic benefits may have attenuated the association between unemployment and psychological distress.^16,56^ However, we adjusted for assets, particularly measures of savings, debt, and income, that inform access to health-promoting resources^57^ and that served as eligibility cutoffs for receipt of federal stimulus checks, thereby mitigating this concern somewhat. Fourth, our exposure definition was broad, grouping household and personal experience of both job loss and income loss from March 24 through August 16, 2020. Further work is needed to identify how income or job loss at different parts of the pandemic were associated with mental health.

## Conclusions

In this cohort study, we aimed to close a gap in the literature on the longer-term influence of income or job loss on mental health in US working-age adults several years after the start of the COVID-19 pandemic and found evidence of a small, but statistically significant association between early-phase pandemic income or job loss and psychological distress more than 2 years later. Efforts to maintain employment and financial stability may help to mitigate the ill effects of job loss in future large-scale events. While the federal Public Health Emergency for COVID-19 has ended, the stressors felt during the pandemic may live on, requiring attention and resources to support ongoing population mental health.

## Notes

Supplement 1.
eMethods.

eResults.
**eFigure 1.** Flow Chart of Sample Inclusion and Survey Measures **eFigure 2.** Distribution of Propensity Scores by Income or Job Loss Group **eFigure 3.** Distribution of Propensity Score Weights Among Those Who Did Not Experience Income or Job Loss **eTable 1.** Demographic Characteristics, Prepandemic Finances, Lifetime Diagnosis of Mental Health Conditions, and Psychological Distress, by Missing Data Status **eTable 2.** Propensity Score Model and Covariate Balance **eTable 3.** Estimated Association Between Early-Pandemic Income or Job Loss and Pandemic Psychological Stress Score
eReferences

Supplement 2.
Data Sharing Statement
