# Supplementary material for: Income or Job Loss and Psychological Distress During the COVID-19 Pandemic
Source: JAMA Netw Open. 2024 Jul 30;7(7):e2424601. doi: 10.1001/jamanetworkopen.2024.24601 (PMC11289696; doi:10.1001/jamanetworkopen.2024.24601)
Supplement: Supplement 1. — eMethods. eResults. eFigure 1. Flow Chart of Sample Inclusion and Survey Measures eFigure 2. Distribution of Propensity Scores by Income or Job Loss Group eFigure 3. Distribution of Propensity Score Weights Among Those Who Did Not Experience Income or Job Loss eTable 1. Demographic Characteristics, Prepandemic Finances, Lifetime Diagnosis of Mental Health Conditions, and Psychological Distress, by Missing Data Status eTable 2. Propensity Score Model and Covariate Balance eTable 3. Estimated Association Between Early-Pandemic Income or Job Loss and Pandemic Psychological Stress Score eReferences [file jamanetwopen-e2424601-s001.pdf]

Supplemental Online Content

Ringlein GV, Ettman CK, Stuart EA. Income or job loss and psychological distress during the COVID-19 pandemic. *JAMA Netw Open*. 2024;7(7):e2424601. doi:10.1001/jamanetworkopen.2024.24601

eMethods.

eResults.

eFigure 1. Flow Chart of Sample Inclusion and Survey Measures

eFigure 2. Distribution of Propensity Scores by Income or Job Loss Group

eFigure 3. Distribution of Propensity Score Weights Among Those Who Did Not Experience Income or Job Loss

eTable 1. Demographic Characteristics, Prepandemic Finances, Lifetime Diagnosis of Mental Health Conditions, and Psychological Distress, by Missing Data Status

eTable 2. Propensity Score Model and Covariate Balance

eTable 3. Estimated Association Between Early-Pandemic Income or Job Loss and Pandemic Psychological Stress Score

eReferences

This supplemental material has been provided by the authors to give readers additional information about their work.

© 2024 Ringlein GV et al. *JAMA Network Open.*

eMethods

We used a sample of participants from the Pew Research Center American Trends Panel (ATP) with data from Wave 54 (September 16-29, 2019) for pre-pandemic financial standing and parental status, Wave 64 (March 19-24, 2020) for pre-income or job loss psychological distress and demographics, Wave 72 (August 3-16, 2020) for income or job loss, Wave 83 (February 16-21, 2021) and Wave 114 (September 13-18, 2022) for post income or job loss psychological distress. Questionnaires for each wave, containing the exact wording of the questions, can be downloaded at https://www.pewresearch.org/american-trends-panel-datasets/.

Survey Methodology

The ATP consists of a nationally representative sample of U.S. non-institutionalized adults who completed periodic surveys ("Waves"). When the ATP began in 2014, recruitment was conducted via random telephone number dialing, but as of 2018, participants are recruited via mail with random stratified sampling based on address.[1]

Each wave contains questions pertaining to a variety of topics and particular questions may be repeated across multiple waves and or only asked in a single wave. Demographic information is collected periodically, but not in every wave[2]. All questions are multiple choice.[1]

Panelists receive notification of the survey via email, SMS, or mail (by panelist preference) and complete the survey over the internet (with tablet and internet connection access available on request for those without internet access), in either English or Spanish.[1] Panelists receive differential incentives to complete surveys based on the relative difficulty of obtaining data from different subpopulations.[1]

Out of 7,347 invited panelists, 6878 (93.6%) completed Wave 54 survey in September 16-29, 2019.[3] During Wave 64 (March 19-24, 2020) 11,537 out of 15,433 invited panelists (74.8%) completed the survey.[4] Between August 3 and 16, 2020, 13,200 out of 15,387 invited panelists (85.8%) completed the Wave 72 survey.[5] Between February 16 and 21, 2021, 10,121 out of 11,605 invited panelists (87.2%) completed the Wave 83 survey.[6] During Wave 114 (September 13-18, 2022), 10,588 out of 11,687 invited panelists (90.6%) completed the survey.[1] There were N=3335 individuals who completed all five of these waves.

Survey Weighting

The Pew Research Center created customized survey weights calibrated for our sample, based on the weights for our baseline wave (Wave 54), adjusted for selection and non-response at the subsequent waves, and trimmed at the 1st and 99th percentiles.[3] Survey weights align the sample with the U.S. population of adults ages 18-64 years who did not experience household income or job loss prior to March 24, 2020. The estimates of the source population for Wave 54 primary came from the 2017 American Community Survey.[1]

Missing data

Where possible, missing demographic data and income tiers were imputed using the closest non-missing time point out of the five waves analyzed. There was no missingness for census region or metropolitan area variables. After imputation using available data from the closest wave in time, there was no missingness in age, sex, or education. Some missingness remained in race and ethnicity, marital status, citizenship, income tier. Lifetime diagnosis of mental illness, parental status, financial strain, pre-pandemic employment, savings account, and debt were not imputed given that they were only collected at one timepoint.

Sample definition

Of the N=3335 who completed all five waves of interest, we excluded n=794 (23.8%) who were aged 65 or older. Of the remaining 2541 participants, the n=796 (31.3%) who reported "Yes, has happened" to having experienced self or household income or job loss due to the pandemic by March 2020 were excluded. Next, of the remaining 1745 participants, we excluded n=272 (15.6%) who reported self or household income or job loss as "Not applicable" in August 2020, leaving 1473 participants. Finally, we excluded participants missing data for self or household income loss (n=1 ,<1%), for any of the mental health measures (anxiety, depression, loneliness, lack of

© 2024 Ringlein GV et al. *JAMA Network Open.*

hope, sleep problems) used to construct psychological distress (n=37, 2.5%), or for any of the covariates (n=43, 2.9%). This defined our analytic sample consisting of N=1392 working aged adults in the U.S.

Measure descriptions

Income or job loss. In Wave 64 (March 2020), the survey questionnaire asked all participants: “For each of the following, indicate whether or not it is something that happened to YOU OR SOMEONE IN YOUR HOUSEHOLD because of the coronavirus outbreak: a. Been laid off or lost a job, b. Had to take a cut in pay due to reduced hours or demand for your work.” The response options were “Yes, has happened” and “No, has not happened.” In August 2020, the questionnaire first asked all participants: “For each of the following, indicate whether or not it is something that happened to YOU because of the coronavirus outbreak: a. Been laid off or lost a job, b. Had to take a cut in pay due to reduced hours or demand for your work.” The response options were “Yes, has happened,” “No, has not happened,” and “Not applicable, not employed”. Then, participants who had indicated that their household size included more than one adult besides themselves were asked “What about other people currently living in your household? Because of the coronavirus outbreak has ANYONE ELSE in your immediate household...” with the same options and responses available. Therefore, in our combined measure of self or household income or job loss, we coded participants as “Not applicable” if they either reported “Not applicable (not employed)” for both measures of self-income or job loss and their values for household income or job loss were both missing, or if they reported “Not applicable (not employed)” for all four measures. If a participant reported “Not applicable (not employed)” for one measure of self-income or job loss but “Yes, has happened” or “No, has not happened” for the other measure of self-income or job loss, we considered the other response to override the “Not applicable (not employed)” response. The same was done for household income or job loss, and the two measured were combined to create our composite measure which we refer to as income or job loss for parsimony.

Psychological Distress
Participants were asked “In the past 7 days, how often have you... a. Felt nervous, anxious, or on edge? b. Felt depressed? c. Felt lonely? d. Felt hopeful about the future? e. Had trouble sleeping?” The response choices were “Rarely or none of the time (less than 1 day)”, “Some or a little of the time (1-2 days)”, “Occasionally or a moderate amount of time (3-4 days)”, and “Most or all of the time (5-7 days)”, coded as 0, 1, 2, and 3 respectively (with hopefulness reverse coded). The composite psychological distress score was created by summing the scores on the five domains. The maximum possible score would be 15, corresponding to reporting “Most or all of the time (5-7 days)” for each of the five domains. The minimum possible score would be 0, corresponding to reporting “Rarely or none of the time (less than 1 day)” for each of the five domains.

Lifetime diagnosis of a mental health condition. In March 2020, participants were asked whether a doctor or other health care provider had ever told them they had a mental health condition.

Pre-pandemic finances and employment: We used the following variables, reported in September 2019: an indicator of secure debt (mortgage/home loans or car loans), an indicator of unsecured debt (credit card, student loans or medical debt), an indicator of financial strain (worrying about bills every day or almost every day), income tier (reported by the Pew Research Center, based on family income adjusted for household size and cost-of-living), and employment status (full-time, part-time, or not employed). Information on retirement status was not available.

Demographics: We used sex, age, race and ethnicity, marital status, education level, citizenship, census region, a metropolitan area indicator, and parental status as demographic covariates. Sex was reported as a binary (male or female) variable. Race and ethnicity were reported as a combined, mutually exclusive construct in four categories: “White non-Hispanic”, “Black non-Hispanic”, “Hispanic” and “Other”. Due to the small number of participants reporting their marital status as “Separated,” we created a composite category representing both separated and divorced participants. March 2020 (Wave 64) data was used for all demographics, except parental status (whether the participant was a parent to a child under the age of 18) which was only reported in September 2019.

Statistical model choice

We modelled the relationship between income or job loss and psychological distress using quasi-Poisson models. This model uses more flexible assumptions on the mean-variance relationship relative to a standard Poisson model and is appropriate for our outcome which consists of non-negative integer values with a skewed right skewed distribution.[7–9]

© 2024 Ringlein GV et al. *JAMA Network Open.*

The quasi-Poisson model assumes a functional form for the conditional mean with a log-link and that the conditional variance is proportional to the conditional mean, allowing for over- or under-dispersion (meaning that the variance is not equal to the mean, as a traditional Poisson model assumes).[7] Although quasi-Poisson models are most commonly used to model counts of events, they can also be used with non-negative integer data or even continuous positive data.

While ordinary least squares (OLS) models are the most common choice for modelling psychometric data, quasi-Poisson models have also been used in the literature[11–15]. For data that is truly distributed as a Poisson with a relatively low mean, OLS may result in biased standard errors.[9,16] Another possible model for this data would be a OLS model with a log transformation of the outcome, however, these models have been show to perform poorly under heteroskedasticity[10] and when some proportion of our data is zero-valued.[17]

Although we use the quasi-Poisson model in our main analysis, given the prevalence of OLS models in the literature, we also conduct a supplemental analysis replacing the quasi-Poisson model with an OLS model (see **eResults** below).

© 2024 Ringlein GV et al. *JAMA Network Open.*

eResults

Propensity score and weight distribution

As described in the main text, we computed propensity scores and corresponding weights using logistic regression, with demographics, pre-pandemic financial status, life-time diagnosis of a mental illness, and baseline psychological distress included in the model to estimate the probability of experiencing income or job loss between Match 24, 2020 August 16, 2020.

We graphically examined the distribution of propensity scores in the two groups to check for common support (**eFigure 2**) and saw overlap between the groups across approximately the entire range of propensity scores in the sample. Propensity scores ranged from 0.11-0.71 (median=0.34) and 0.11-0.79 (median=0.31) in the groups who did and did not report income or job loss, respectively.

To weight our sample to the group who experienced income or job loss, those in this group are assigned a propensity score weight of 1, and those who did not experience income or job loss were weighted by their odds of experiencing income or job loss. These weights ranged from 0.13-3.85 (median=0.46) in the group who did not report income or job loss, as seen in **eFigure 3**. Based on this qualitative evaluation we did not consider any of these weights to be extreme or require trimming.

Analysis using an OLS model

To investigate the robustness of our findings, we repeated the main analysis using an ordinary least squares model instead of a quasi-Poisson. We estimate that on a difference in means scale, early phase pandemic income or job loss was associated increases of 0.42 (95% CI=0.00-0.84, p=0.05) and 0.52 (95% CI=0.05-0.98, p=0.03) points higher, respectively. These estimates and CIs are of similar magnitude to the quasi-Poisson estimates and support the conclusions discussed in the main text.

© 2024 Ringlein GV et al. *JAMA Network Open.*

eFigure 1. Flow Chart of Sample Inclusion and Survey Measures

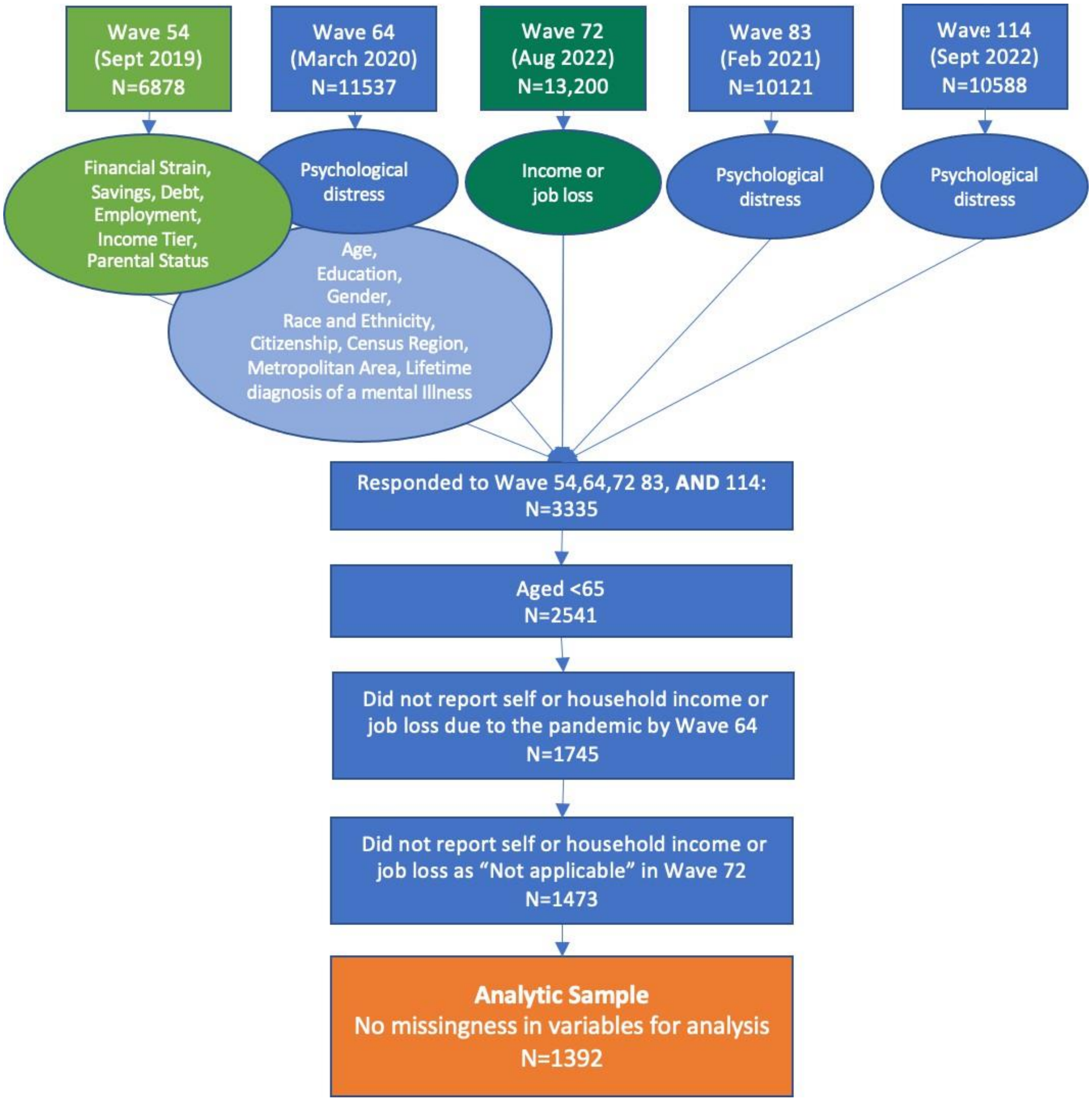

eFigure 1: Using data from Waves 54, 64, 72, 83, and 114 of the of Pew Research Center's American Trends Panel data, we subset our sample to only those who responded to all five waves, were younger than age 65, did not report self or household income or job loss due to the pandemic by March 2020, did not report reported self or household income or job loss as "Not applicable" in August 2020, and were not missing data on the variables of interest.

© 2024 Ringlein GV et al. *JAMA Network Open.*

eFigure 2. Distribution of Propensity Scores by Income or Job Loss Group

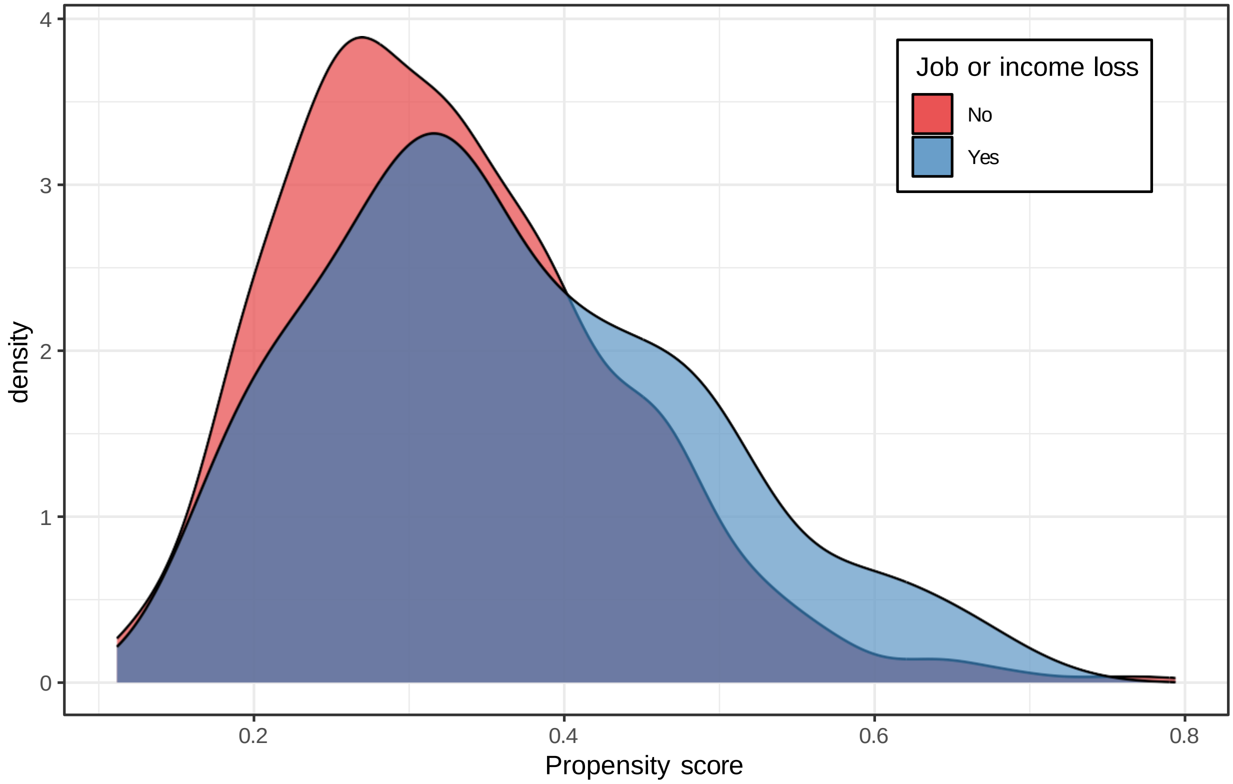

eFigure 2: The distribution of propensity scores (estimated probability of experiencing income or job loss between March 24, 2020 and August 16, 2020) is shown among the groups who did and did not experience income or job loss, in our sample of adults 18-64 years in the U.S. who had not experienced income or job loss by March 24, 2020 (N=1392). Data comes from the of Pew Research Center's American Trends Panel data.

eFigure 3. Distribution of Propensity Score Weights Among Those Who Did Not Experience Income or Job Loss

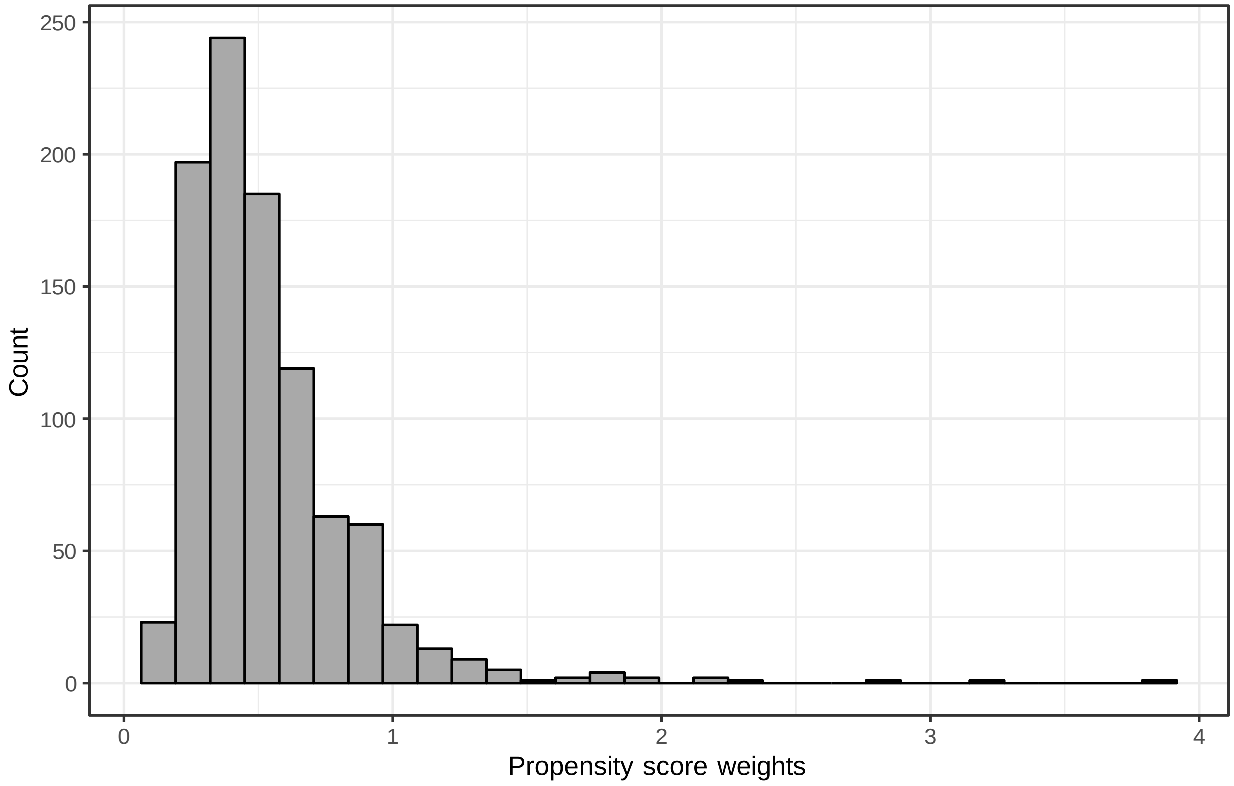

eFigure 3: A histogram of propensity score weights is shown for those who did not report income or job loss by August 16, 2020 (n=955). Those who did report income or job loss between March 24, 2020 and August 16, 2020 are assigned weights of one. Data comes from the of Pew Research Center’s American Trends Panel data.

© 2024 Ringlein GV et al. *JAMA Network Open.*

eTable 1. Demographic Characteristics, Prepandemic Finances, Lifetime Diagnosis of a Mental Health Condition, and Psychological Distress, by Missing Data Status[a]

Characteristic	Missing data, No. (%)[b]	No missing data, No. (%)[b]
Full analytic sample and missing data sample	81 (6.4)	1392 (93.6)
Mid-pandemic income or job loss		
No, has not happened	51 (62.0)	955 (64.2)
Yes, has happened	29 (38.0)	437 (35.8)
Missing	12	0
Sex		
Male	33 (44.9)	694 (52.7)
Female	48 (55.1)	698 (47.3)
Age Category		
18-29	17 (29.1)	258 (24.6)
30-49	32 (41.1)	638 (47.7)
50-64	32 (29.8)	496 (27.8)
Race and Ethnicity		
White non-Hispanic	48 (51.7)	971 (63.0)
Black non-Hispanic	12 (13.2)	130 (11.8)
Hispanic	10 (14.6)	188 (15.6)
Other	8 (20.5)	103 (9.6)
Missing	1	0
Marital Status		
Married	44 (42.3)	807 (55.9)
Divorced or separated	10 (13.9)	144 (9.6)
Living with a partner	3 (4.7)	125 (10.1)
Widowed	1 (1.3)	28 (1.2)
Never been married	22 (37.8)	288 (23.2)
Education Category		
Less than high school	1 (6.0)	31 (5.5)
High school graduate	20 (31.7)	201 (21.9)
Associate’s degree	6 (4.8)	138 (9.3)
Some college	14 (20.7)	295 (24.2)
College graduate+	20 (21.0)	421 (22.6)
Postgraduate	20 (15.9)	306 (16.5)
Parent of a child under 18	26 (32.3)	489 (35.8)
Missing	1	0
U.S. Citizenship	79 (95.7)	1,350 (94.2)
Census Region		
South	37 (47.7)	524 (39.8)
Northeast	15 (23.7)	221 (16.4)
Midwest	16 (19.5)	317 (20.6)
West	13 (9.0)	330 (23.3)
Metropolitan Area	75 (93.2)	1,234 (87,3)
Income Tier (Pre-pandemic)		
Upper income	22 (25.3)	415 (23.3)
Lower income	12 (18.6)	251 (25.7)
Middle income	35 (56.1)	726 (50.9)
Missing	14	0
Financial Strain (Pre-pandemic)	20 (33.4)	450 (37.7)
Missing	15	0
Employment Status (Pre-pandemic)		
Full-time	53 (60.5)	976 (64.9)

© 2024 Ringlein GV et al. *JAMA Network Open.*

Part-time	8 (11.8)	163 (12.8)
Not employed	16 (27.7)	253 (22.4)
Missing	3	
Had a Savings Account (Pre-pandemic)	54 (72.4)	1,130 (74.7)
Missing	17	0
Had Secure Debt (Pre-pandemic)	58 (72.2)	989 (65.4)
Missing	7	0
Had Unsecured Debt (Pre-pandemic)	46 (63.5)	941 (68.1)
Missing	7	0
Lifetime Diagnosis of a Mental Health Condition	8 (16.8)	222 (16.0)
Missing	12	0
	**Mean (SD)** [c]	**Mean (SD)** [c]
Psychological Distress in March 2020 [d]	5.0 (3.8)	5.1 (3.4)
Missing	19	0
Psychological Distress in February 2021[d]	5.4 (4.1)	4.9 (3.4)
Missing	12	0
Psychological Distress in September 2022 [d]	5.0 (3.6)	5.0 (3.5)
Missing	18	0

[a] The missing data sample consists of participants who are missing data for at least one covariate, psychological distress measurement, or early-pandemic income or job loss. The sample of participants not missing data is the analytic sample used in the main text, which consists of U.S. adults aged 18-64 who did not experience income or job loss before March 24, 2020 from the Pew Research Center's American Trends Panel.
[b] Number of participants (No.) is unweighted, percentage (%) is weighted with longitudinal survey weights. Missing data is excluded for in percentage calculations to facilitate comparison between groups.
[c] Mean and standard deviation are weighted with longitudinal survey weights. Missing data is excluded for in percentage calculations to facilitate comparison between groups.
[d] Psychological distress measured on a composite scale from 0-15 based on participants' reported frequency of feeling depressed, on edge, sleepless, lonely, and hopeless in the last week). Psychological distress in March 2020 is reported prior to any of this sample experiencing household income or job loss and is used as a covariate in our main analysis. February 2021 and September 2022 psychological distress are the dependent variables of interest in our main analysis.

© 2024 Ringlein GV et al. *JAMA Network Open.*

eTable 2. Propensity Score Model[a] and Covariate Balance

	Propensity score model[a]			Covariate balance (SMD)[b]	
**Characteristic**	**aOR**	**95% CI**	***p*-value**	**Unadjusted**	**Adjusted**
Psychological Distress (March 2020)	1.01	0.96, 1.07	0.7	0.027	-0.029
Lifetime Diagnosis of a Mental Health Condition: Yes	0.82	0.53, 1.29	0.4	-0.027	-0.021
Sex: Female	0.85	0.62, 1.17	0.3	-0.089	-0.012
Age Category					
50-64	—[c]	—	—	-0.135	-0.015
18-29	1.64	1.01, 2.67	0.047	0.178	-0.014
30-49	1.10	0.75, 1.61	0.6	-0.047	0.026
Race and Ethnicity					
White non-Hispanic	—	—	—	-0.166	-0.028
Black non-Hispanic	0.99	0.58, 1.68	>0.9	-0.082	-0.005
Hispanic	1.64	0.99, 2.71	0.056	0.185	0.027
Other	1.61	0.91, 2.83	0.10	0.100	0.014
Marital Status					
Married	—	—	—	-0.041	0.021
Divorced or Separated	1.07	0.63, 1.82	0.8	-0.006	-0.019
Living with a partner	1.13	0.66, 1.94	0.7	0.09	-0.008
Widowed	1.26	0.50, 3.19	0.6	0.009	0.021
Never been married	0.75	0.47, 1.19	0.2	-0.019	-0.011
Education Category					
Postgraduate	—	—	—	-0.068	-0.015
Associate's degree	0.63	0.35, 1.12	0.11	-0.164	-0.006
College graduate+	1.13	0.76, 1.68	0.5	-0.005	-0.023
High school graduate	1.11	0.66, 1.88	0.7	0.079	0.027
Less than high school	1.48	0.59, 3.75	0.4	0.078	0.056
Some college	1.00	0.63, 1.60	>0.9	0.031	-0.021
Parent of a child under 18: Yes	1.04	0.72, 1.51	0.8	0.031	0.008
Citizenship: Not a US citizen	0.77	0.33, 1.80	0.5	0.05	0.024
Census Region					
South	—	—	—	-0.038	-0.005
Northeast	0.72	0.45, 1.15	0.2	-0.199	0.002
Midwest	1.11	0.74, 1.66	0.6	-0.013	0.016
West	1.50	1.00, 2.23	0.048	0.196	-0.010
Metropolitan Area: No	0.79	0.48, 1.31	0.4	-0.054	0.000
Income Tier					
Upper income	—	—	—	-0.200	-0.012
Lower income	1.74	1.03, 2.94	0.039	0.194	0.022
Middle income	1.42	0.98, 2.07	0.065	-0.026	-0.011
Financial Strain: Yes	1.13	0.78, 1.64	0.5	0.127	0.014
Employment Status					
Full-time	—	—	—	-0.038	0.026
Part-time	1.43	0.88, 2.33	0.2	0.149	-0.016
Not employed	0.73	0.47, 1.13	0.2	-0.092	-0.017
Savings Account: Yes	1.06	0.69, 1.63	0.8	0.098	0.021
Secure Debt: Yes	1.01	0.69, 1.47	>0.9	-0.083	0.029
Unsecured Debt: Yes	1.01	0.69, 1.47	>0.9	-0.083	0.029

[a] We used a multivariable survey weighted logistic regression to model the probability of experiencing income or job loss between March 24, 2020 - August 16, 2020. Adjusted odds ratio (aOR), 95% confidence intervals (CI), and *p*-value are reported for each covariate. The analytic sample consists of U.S. adults aged 18-64 who did not experience income or joss before March 24, 2020.
[b] Standardized mean difference (SMD). Unadjusted: Survey weighted only. Adjusted: Survey and propensity score weighted.
[c] A dash indicates a reference level. For two-category variables, reference level is not displayed.

© 2024 Ringlein GV et al. *JAMA Network Open.*

eTable 3. Estimated Association Between Early-Pandemic Income or Job Loss and Pandemic Psychological Stress Score

**Psychological Distress**	**Ratio[a] of average score between groups**	**95% CI for Ratio**	**p-value for Ratio**	**Difference[b] in average score between groups**	**95% CI for Difference**	**p-value for Difference**
Before income or job loss (baseline characteristics, prior to income or job loss)[c]						
March 2020	0.98	0.90, 1.08	0.75	-0.08	-0.56, 0.40	0.75
After income or job loss[d] (outcomes)						
February 2021	1.09	1.01, 1.18	0.03	0.42	0.03, 0.80	0.03
September 2022	1.11	1.02, 1.22	0.02	0.52	0.10, 0.95	0.02

[a] This ratio is obtained by exponentiating the coefficient of the survey and propensity score weighted multivariable quasi-Poisson model and is presented with 95% confidence intervals and *p*-value.
[b] This difference is obtained from the quasi-Poisson models using the marginal effect package in R to compute the point estimate, 95% confidence intervals and *p*-value.
[c,d] Demographics, pre-pandemic finances, life-time diagnosis of a mental health condition are included as covariates in all three models, and psychological distress in March 2020 is included as a covariate for the late-pandemic models. All these covariates are included in the propensity score model.
Data: Pew Research Center's American Trends Panel. Sample: Adults 18-64 years in the U.S. who had not experienced income or job loss by March 24, 2020 (N=1392).

© 2024 Ringlein GV et al. *JAMA Network Open.*

eReferences

1. *Pew Research Center’s American Trends Panel Wave 114 Methodology Report*. Pew Research Center; 2022.

2. *Codebook and Instructions for Working with American Trends Panel Data*. Pew Research Center; 2021. https://www.pewresearch.org/wp-content/uploads/2018/05/Codebook-and-instructions-for-working-with-ATP-data.pdf

3. *Pew Research Center’s American Trends Panel Wave 54 Methodology Report*. Pew Research Center; 2019.

4. *Pew Research Center’s American Trends Panel Wave 64 Methodology Report*. Pew Research Center; 2020.

5. *Pew Research Center’s American Trends Panel Wave 72 Methodology Report*. Pew Research Center; 2020.

6. *Pew Research Center’s American Trends Panel Wave 83 Methodology Report*. Pew Research Center; 2021.

7. McCullagh P, Nelder JA. Log-linear models. In: *Generalized Linear Models*. 2nd ed. Monographs on statistics and applied probability. Chapman & Hall/CRC; 1998:193-208.

8. Wooldridge JM. Count data and Related Models. In: *Econometric Analysis of Cross Section and Panel Data*. MIT Press; 2001:645-678.

9. Coxe S, West SG, Aiken LS. The Analysis of Count Data: A Gentle Introduction to Poisson Regression and Its Alternatives. *J Pers Assess*. 2009;91(2):121-136. doi:10.1080/00223890802634175

10. Silva JMCS, Tenreyro S. The Log of Gravity. *Rev Econ Stat*. 2006;88(4):641-658. doi:10.1162/rest.88.4.641

11. Sacchi C, Girardi P, Buri A, De Carli P, Simonelli A. The perinatal health secondary to pandemic: association between women’s delivery concerns and infant’s behavioral problems. *J Reprod Infant Psychol*. Published online March 17, 2024:1-16. doi:10.1080/02646838.2024.2330662

12. Tampubolon G, Hanandita W. Poverty and mental health in Indonesia. *Soc Sci Med*. 2014;106:20-27. doi:10.1016/j.socscimed.2014.01.012

13. Khosrorad R, Aval HE, Najafi ML, et al. The association of exposure to air pollution and depression in women; a cross-sectional study of a middle-income country. *Environ Res*. 2022;215:114266. doi:10.1016/j.envres.2022.114266

14. Nnodum BN, McCormack MC, Putcha N, et al. Impact of Physical Activity on Reporting of Childhood Asthma Symptoms. *Lung*. 2017;195(6):693-698. doi:10.1007/s00408-017-0049-7

15. Lee S, Kang M, Lee J, Lee HJ. Long-term effects of childhood difficulties on depressive symptoms among older adults: Role of adulthood income and income satisfaction. *Geriatr Gerontol Int*. 2024;24(S1):246-252. doi:10.1111/ggi.14833

16. Gardner W, Mulvey EP, Shaw EC. Regression analyses of counts and rates: Poisson, overdispersed Poisson, and negative binomial models. *Psychol Bull*. 1995;118(3):392-404. doi:10.1037/0033-2909.118.3.392

17. Mullahy J, Norton EC. Why Transform Y? The Pitfalls of Transformed Regressions with a Mass at Zero*. *Oxf Bull Econ Stat*. 2024;86(2):417-447. doi:10.1111/obes.12583

© 2024 Ringlein GV et al. *JAMA Network Open.*
