# Supplementary material for: Income or Job Loss and Psychological Distress During the COVID-19 Pandemic
Source: JAMA Netw Open. 2024 Jul 30;7(7):e2424601. doi: 10.1001/jamanetworkopen.2024.24601 (PMC11289696; doi:10.1001/jamanetworkopen.2024.24601)
Supplement: Supplement 2. — Data Sharing Statement [file jamanetwopen-e2424601-s002.pdf]

Data Sharing Statement

Ringlein. Income or Job Loss and Psychological Distress During the COVID-19 Pandemic. *JAMA Netw Open*. Published July 30, 2024. doi:10.1001/jamanetworkopen.2024.24601

Data
Data available: Yes
Data types: Data (not involving human participants)
How to access data: Data are publicly available on the Pew American Trends Panel (ATP) website: https://www.pewresearch.org/our-methods/u-s-surveys/the-american-trends-panel/
When available: With publication

Supporting Documents
Document types: Statistical/analytic code
How to access documents: Requests for statistical/analytic code can be sent to: gringle1@jhmi.edu. Our code will be made publicly available on github upon publication: https://github.com/gringle1/ps_distress_incomeloss
When available: With publication

Additional Information
Who can access the data: Data are publicly available on the Pew American Trends Panel (ATP) website: https://www.pewresearch.org/our-methods/u-s-surveys/the-american-trends-panel/
Types of analyses: Requests for statistical/analytic code can be sent to: gringle1@jhmi.edu.
Mechanisms of data availability: Data are publicly available on the Pew American Trends Panel (ATP) website: https://www.pewresearch.org/our-methods/u-s-surveys/the-american-trends-panel/ Our code will be made publicly available on github upon publication: https://github.com/gringle1/ps_distress_incomeloss
